# Availability and Diversity of Training Programs for Responders to International Disasters and Complex Humanitarian Emergencies

**DOI:** 10.1371/currents.dis.626ae97e629eccd4756f20de04a20823

**Published:** 2014-06-23

**Authors:** Gabrielle A. Jacquet, Chioma C. Obi, Mary P. Chang, Jamil D. Bayram

**Affiliations:** Department of Emergency Medicine, Boston University School of Medicine, Boston, Massachusetts, USA; Center for Global Health and Development, Boston University, Boston, Massachusetts, USA; Department of International Health, Johns Hopkins Bloomberg School of Public Health, Baltimore, Maryland, USA; Department of Emergency Medicine, Johns Hopkins University School of Medicine, Baltimore, Maryland, USA; Johns Hopkins University, Baltimore, Maryland, USA; Johns Hopkins Office of Critical Event Preparedness and Response, Baltimore, Maryland, USA; Center for Refugee and Disaster Response, Bloomberg School of Public Health, Johns Hopkins University, Baltimore, Maryland, USA

**Keywords:** complex humanitarian emergencies, disaster, pre-deployment, preparedness, training programs

## Abstract

Introduction: Volunteers and members of relief organizations increasingly seek formal training prior to international field deployment. This paper identifies training programs for personnel responding to international disasters and complex humanitarian emergencies, and provides concise information – if available- regarding the founding organization, year established, location, cost, duration of training, participants targeted, and the content of each program. Methods: An environmental scan was conducted through a combination of a peer-reviewed literature search and an open Internet search for the training programs. Literature search engines included EMBASE, Cochrane, Scopus, PubMed, Web of Science databases using the search terms “international,” “disaster,” “complex humanitarian emergencies,” “training,” and “humanitarian response”. Both searches were conducted between January 2, 2013 and September 12, 2013. Results: 14 peer-reviewed articles mentioned or described eight training programs, while open Internet search revealed 13 additional programs. In total, twenty-one training programs were identified as currently available for responders to international disasters and CHE. Each of the programs identified has different goals and objectives, duration, expenses, targeted trainees and modules. Each of the programs identified has different goals and objectives, duration, expenses, targeted trainees and modules. Seven programs (33%) are free of charge and four programs (19%) focus on the mental aspects of disasters. The mean duration for each training program is 5 to 7 days. Fourteen of the trainings are conducted in multiple locations (66%), two in Cuba (9%) and two in Australia (9%). The cost-reported in US dollars- ranges from $100 to $2,400 with a mean cost of $480 and a median cost of $135. Most of the programs are open to the public, but some are only available by invitation only, such as the International Mobilization Preparation for Action (IMPACT) and the United Nations Humanitarian Civil-Military Coordination (UN-CMCoord) Field Course. Conclusions: A variety of training programs are available for responders to disasters and complex humanitarian emergencies. These programs vary in their objectives, audiences, modules, geographical locations, eligibility and financial cost. This paper presents an overview of available programs and serves as a resource for potential responders interested in capacity-building training prior to deployment.

## INTRODUCTION

The competence of responders to international disasters is one of the cornerstones for successful program interventions. Hence, building capacity for responders before deployment is an investment to their organizations.^1^^,^ 2 International relief efforts depend heavily on volunteers when responding to international disasters, and volunteers look for structured training programs as an opportunity to build their capacity and to be better prepared.^3^ The total percentage of volunteer responders working with non-governmental organizations (NGO) in response to international disasters and complex humanitarian emergencies is not well documented in the literature. Adequately trained responders not only provide better service, but also present a smaller risk for organizations and a higher level of operational efficiency.

When responding to international disasters, most health care professionals face austere environments with limited resources. Some volunteers are organized in advance and have been trained and directed to respond through government programs (e.g., Disaster Medical Assistance Teams [DMAT], Medical Reserve Corps [MRC]) and private sector efforts (e.g., American Red Cross, Orthopedic Trauma Association Mass Casualty Teams [OTAMCT]).^4^^,^ 5 ^,^ 6 There are also “spontaneous volunteers” who show up ready to help but lack organization, identification, credentials, and, ultimately, utility. Rather than assisting in the emergency efforts, the presence of many uncoordinated volunteers can actually impede effective emergency responses and create a liability to organizations and to themselves.^2^ The most vulnerable responders are novice spontaneous volunteers who lack the experience going on their own instead of through an organization. They may find themselves in situations that require heightened security awareness, cultural sensitivity, and behavioral mental health skills. The better prepared a volunteer is to fill his or her role, the smaller the chance of unintended harm. Volunteers need to know not only what they should do but also what they should not do. As part of this strategy, organizations should make available volunteer position descriptions, a code of conduct, and appropriate training courses and exercises.^7^

In addition, some volunteers need specialized training in caring for pediatric patients or patients who are suffering from mental health issues. Children are often the worse victims of disasters, with the under-5 mortality rate approaching 13% in a typical 5-year war.^8^^,^ 9 They require specialized medical care as well as intense psychological support.^10^

The exact number of responders to international disaster and Complex Humanitarian Emergencies (CHEs) is not well documented in the literature, nor is the percentage that have received formal structured training before actual response. Many organizations do not formally train their own personnel before deployment, let alone volunteers.^11^ This is perhaps the case because identification and characterization of various structured training programs and opportunities available to responders to international disasters is lacking. The objective of this manuscript was to identify and compile a list of the various structured training programs available for responders to international disasters and complex humanitarian emergencies, highlighting their diverse scopes and characteristics.

## METHODS

An environmental scan was conducted through a peer-reviewed literature search of PubMed, Scopus, EMBASE, Web of Science, Cochrane databases, and an open Internet search for training program websites. The landscape literature search was conducted by combining the search terms “international,” “disaster,” “complex humanitarian emergencies,” “training,” and “humanitarian response.” All searches were conducted from January2, 2013 to September 12, 2013. Initially, 681 citations were retrieved from the above named search engines. Screening by titles narrowed the focus to 165 articles and a further screening by abstract led to 40 articles, of which a full text review revealed 14 articles that contained information regarding training programs for responders to international disasters and CHE.^8^^,^ 11 ^,^ 12 ^,^ 13 ^,^ 14 ^,^ 15 ^,^ 16 ^,^ 17 ^,^ 18 ^,^ 19 ^,^ 20 ^,^ 21 ^,^ 22

**Program Identification Method:**
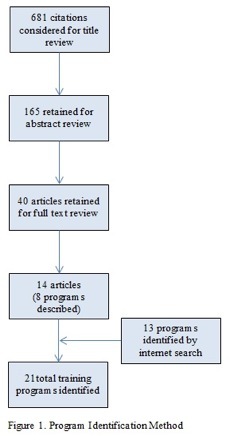

## RESULTS

A total of 14 peer-reviewed articles mentioned or described eight training programs, while open Internet search revealed 13 additional programs. In total, twenty-one training programs were identified as currently available for responders to international disasters and CHE.

Each of the programs identified has different goals and objectives, duration, expenses, targeted trainees and modules. Seven programs (33%) are free of charge and four programs (19%) focus on the mental aspects of disasters. The mean duration for each training program is 5 to 7 days. Fourteen of the trainings are conducted in multiple locations (66%), two in Cuba (9%) and two in Australia (9%). The cost-reported in US dollars- ranges from $100 to $2,400 with a mean cost of $480 and a median cost of $135. Most of the programs are open to the public, but some are only available by invitation, such as the International Mobilization Preparation for Action (IMPACT) and the United Nations Humanitarian Civil-Military Coordination (UN-CMCoord) Field Course. The target audience for IMPACT should satisfy three conditions: 1) have met the established criteria (technical competency in an international emergency response context in one of the “core functions”, “field experience”, and available for four-week voluntary deployment), 2) successfully completed screening interviews, and 3) completed World of Red Cross (WORC) training program.^23^ UN-CMCoord Field course participants should satisfy the following five conditions: 1) completed the UN-CMCoord course, 2) have a minimum of five year experience with civil-military coordination in the humanitarian assistance, 3) fluency in oral and written English; 4) fluency in other UN language and 5) currently performing a task that require interaction with the Military.^24^

Table 1 displays the twenty-one structured training programs (in alphabetical order), along with the organizer, location(s), cost, duration, and year established.

**Table 1: Training Programs:** 

Name	Organizer	Type of Participants	Location	Cost (USD)	Duration	Year Established
ADPC	NI	Government officials responsible for disaster management plan and policy; personnel involved in defense forces and emergency services	Thailand	$2,275-3,175	2 weeks	1986
CDAC	Australia Red Cross	Red Cross staff, volunteers; anyone interested in building international capacity	Australia	$650-920	2 days	NI
CDHRTP	University of Toronto Koffler Scientific Reserve	Anyone interested in humanitarian work	Canada	$2,080	2 weeks	NI
CHART	ICRC	Civilian and military disaster planners	Multiple	NI	5 days	1993
CHR	Cuba	Not reported	Cuba	Free	Rolling	2005
DHMP	American Red Cross, FEMA	Professionals from diverse mental health backgrounds	Multiple	$500	2 days	1990
DMTC	Rainbow Center for International Child Health	Health care professionals and physicians	Multiple	Free	5 days	1996
ELAM	Cuba	Anyone interested in disaster preparedness	Cuba	Free	6 years	1999
HELP	ICRC	Medical and public health professionals, environmental health engineers, and epidemiologists	Multiple	$1,800	2-3 weeks	1986
IDM	Indian Government	Undergraduate and post-graduate students in Symbiosis International University (SIU), India	SIU India	Free	18 months	2005
IHAT/IHPT	Australia Red Cross	Humanitarian aid workers, program officers/coordinators, caseworkers, policy officers or others working with people affected by disaster	Australia	$650-850	3 days	NI
IHE	RedR	Health workers and any professional groups who wish to work in emergency relief	Multiple	$600-2,500	5-7 days	1980
IMPACT	IFRC	By invitation only	Multiple	Free	2 weeks	NI
MIHA, IDHA, ICTC, DMTC, MHCE	CHIC	Members of international relief organizations and humanitarian workers	Multiple	$1,000-5,000	1,2,4 weeks	1992
PHCE	IRC & WE Inc.	NGO staff who are responsible for making decisions that affect the health of refugees, internally displaced persons, and those affected by CHE; District Medical Officers and other Ministry of Health staff working in regions affected by complex emergencies	Multiple	$2,400	2 weeks	NI
ROC	Medair	Mandated for prospective Medair employees but open to the public	Europe & Asia	$750	7 days	NI
TAP	ICRC	Civilian and military disaster planners	Multiple	NI	2-4 days	1980
UN-CMCoord Course and Field Course	OCHA	Governmental and non-governmental organizations, aid agencies, civil protection units, military and civil defense organizations, UN agencies and other intergovernmental bodies, the Red Cross, and Red Crescent Movement	Multiple	Free	2-7 days	1998
Various training programs	Salvation Army	Spiritual care officers, disaster workers trained in critical incident stress debriefing, chaplains, and mental health professionals	Multiple	$135-$350	4-8 hours to 3 days	2004
Various training programs	ICISF	People working in the following fields: crisis intervention, disaster response, education, emergency services, employee assistance, healthcare, homeland security, human resources, mental health, military, spiritual care, transportation, and traumatic stress	Multiple	$100-$700	2-3 days per course	1989
WORC	IFRC	Red Cross and Red Crescent staff, volunteers, government authorities, donors, media, schools, and interested public	Multiple	Free	1 week	NI

**Table 1 abbreviations:**

ADPC= Asian Disaster Preparedness Center; CDAC= Capacity Development across Cultures; CDHRTP= Canadian Disaster and Humanitarian Response Training Program; CHART= Combined Humanitarian Assistance Response Training; CHR= Contingency Henry Reeve Program; CIHC =Center for International Humanitarian Cooperation DMTC= Disaster Management Training course; DMHP= Disaster Mental Health Program by the American Red Cross. ELAM Escuela Latino Americana de Ciencias Médicas (in Spanish; in English Latin American Medical School); FEMA= Federal Emergency Management Agency; HELP= Humanitarian Emergencies for Large Populations; IHAT= International Humanitarian Action Training; IHPT= International Humanitarian; Protection Training; IDM= Integrated Disaster Management Program; IMPACT= International Mobilization and Preparation for ACTion; ICISF= International Critical Incident Stress Foundation; ICRC= International Committee of the Red Cross; IDHA= International Diploma in Humanitarian Assistance; IFRC= International Federation of Red Cross and Red Crescent Societies; ICTC=In country training course; IHE= International Health Exchange programs; IRC= International Rescue Committee; MIHA=Masters in International Humanitarian Action; MHCE=Mental Health in Complex Emergencies; NI=No information; OCHA= United Nations Office for the Coordination of humanitarian Affairs; PHCE= Public Health Complex Emergencies ROC= Recovery Orientation Course, SIU= Symbiosis International University TAP= Training assistance program; UN-CMCoord= Humanitarian Civil-Military Coordination WE Inc.= World Education, Inc. WORC= World of Red Cross; Yr. Est.= year established.

Below are descriptions of the objectives, target audience, course size, and training content of the twenty structured training programs listed in the table above. Information is listed alphabetically and depends on its availability from the various sources queried.

**Asian Disaster Preparedness Center (ADPC)** 21 : The objective is to develop participants’ skills with the aim that they should gain a solid grasp of disaster management processes and be able to address key implementation issues in disaster. Typical participants include government officials responsible for disaster management plan and policy; and personnel involved in defense forces and emergency services. There is a maximum of 30 participants per course. The main topics include disaster preparedness planning and disaster risk reduction.^25^

**Capacity Development Across Cultures (CDAC)**: The objectives include developing skills for building relationships, understanding capacity, and working collaboratively with partners and colleagues to achieve capacity development outcomes. The participants are Red Cross staff, volunteers, and anyone interested in building capacity internationally. There is no course size restriction.^26^ The main topics are not delineated in details.

**Canadian Disaster and Humanitarian Response Training Program (CDHRTP)**: The objectives include developing capacity for essentials during a humanitarian response, managing acute issues in complex disasters, and learning research skills. This course also places participants in a three-day simulation complex humanitarian emergency to train their capacity building and problem solving skills. The participants are anyone interested in humanitarian work. There is no course size restriction listed. Topics include gender based violence, leadership, human rights, monitoring and evaluation, and, refugee protection.^27^

**Combined Humanitarian Assistance Response Training (CHART)** 11 ^,^ 28: The objective is to develop civilian and military response to humanitarian crises in a more efficient manner. It is designed to provide responders with the tools and knowledge to work effectively in rapidly evolving situations. Participants are civilian and military disaster planners, and the maximum number of participants admitted per course is sixty. The main subjects include the complex environment, security, logistics, nutrition, public health, international standards, responder readiness, planning, peacekeeping issues, and international humanitarian law.^29^

**Contingency Henry Reeve Program (CHR)**: The objective is to develop responders who can aid in saving lives, especially in remote areas. The training program focuses on delivering primary care in disasters and epidemics. The type of participants is not documented. There are no restrictions to number of participants. The main topics includes disaster medicine courses for all types of emergencies.^30^

**Disaster Mental Health Program (DMHP)**: The objective is to develop resilience among responders by ameliorating acute post disaster stress responses among Red Cross workers and the disaster victims they serve. Participants are comprised of professionals from diverse mental health backgrounds. The number of participants and course titles are not delineated.^31^

**Disaster Management Training Course (DMTC)**: The specific objectives are to develop participant understanding as to why children are among the most vulnerable populations in disasters and to identify the most important problems and priorities. The program targets health care professionals and physicians as participants. There is no restriction to the number of participants. Lecture topics included epidemiological assessment, triage during disasters, and malnutrition.

**Escuela Latino Americana de Ciencias Médicas (ELAM)** 14: The objective is for participants to develop skills necessary for disaster response. The program has a strong clinical component. Participants worldwide are welcome if they have an interest in disaster preparedness. There are no restrictions to the number of participants. The main topics include disaster medicine courses for all types of emergencies.^30^

**Humanitarian Emergencies for Large Populations (HELP)** 11 ^,^ 12 ^,^ 16 ^,^ 32: This course is a multicultural and multidisciplinary learning experience created to enhance professionalism within humanitarian assistance programs in the setting of disasters and CHE. Participants are medical and public health professionals, environmental health engineers, and epidemiologists. Twenty-five to thirty participants are admitted per course. The main topics include public health activities, communicable diseases, epidemiology, international humanitarian law (IHL), security, humanitarian principles, and ethics.^33^

**Integrated Disaster Management (IDM)**: The objectives are to ensure and develop awareness of the nature, type, and management of disasters, design disaster management plans as well as hands-on training in handling medical and non-medical emergencies. The participants are undergraduate and post-graduate students in Symbiosis International University (SIU), India. The number of participants and main topics were not outlined.^15^

**International Humanitarian Action Training (IHAT)/ International Humanitarian Protection Training (IHPT).** **IHAT:** The objective is to develop and equip individuals and organizations with the knowledge needed to respond effectively and implement recovery after disasters. It is open to current and aspiring aid workers, volunteers, and those interested in international emergency response. There are no restrictions to the number of participants per course.^26^ The main topics were not listed. **IHPT:** The objective is to develop understanding and competencies for protection into humanitarian action (Sphere 2011) in four key areas: child protection, gender-based violence (GBV), sexual exploitation and abuse (SEA), and other human rights abuses. Participants are humanitarian aid workers, program officers/coordinators, caseworkers, policy officers or others working with people affected by disaster. There are no restrictions to the number of participants per course. The IHAT course is a pre-requisite to this course.^34^ The main topics include gender-based violence (GBV) and child protection among others.

**International Health Exchange programs (IHE)**: The objective is for aid workers around the world to develop the latest skills to ensure the quality and effectiveness of humanitarian programs. The participants are health workers and any professional groups who wish to work in emergency relief. There are no restrictions to number of participants per course. The main topics include humanitarian essentials, security, and technical training.^35^

**International Mobilization and Preparation for ACTion (IMPACT)** 19: The objective is to provide additional training to participants by taking an in-depth look at the roles of the ICRC, the Federation, and National Societies in times of disaster and conflict. Another objective is to provide contextual and capacity building skills for relief and development activities. Participation is by invitation only and limited to 20 applicants. Interested parties who meet the established criteria, successfully complete screening interviews, reference checks, and WORC? may be invited to participate in the course. The course covers practical subjects such as dealing with stress, security, and cross-cultural awareness.^23^

**Masters in International Humanitarian Action (MIHA & OTHER PROGRAMS)**: Center for International Humanitarian Cooperation (CHIC) offers several humanitarian training programs. The objective is for humanitarian workers and members of international relief agencies to perform more effectively in conflict and post-conflict areas. The CHIC designs training courses tailored to organizations or geographical location. Participants are members of international relief organizations and humanitarian workers. There are no restrictions to the number of participants per course. Main topics include mental health issues, disaster management, and negotiation techniques.^36^

**Public Health Complex Emergencies (PHCE)** 12: The objectives are to develop and sharpen practical problem-based skills in an interactive group setting and to help participants become well-informed decision-makers and managers of public health policy in complex emergencies. Participants are non-governmental organization (NGO) staff who are responsible for making decisions that affect the health of refugees, internally displaced persons, and those affected by CHE. Other participants include District Medical Officers and other Ministry of Health staff working in regions affected by complex emergencies. There are no restrictions to the number of participants per course. The main topics include context of emergencies, reproductive health, epidemiology psychosocial health, communicable disease weapons, violence and trauma, and environmental health protection and security and nutrition coordination.^37^

**Recovery Orientation Course (ROC)**: The ROC uses an intensive experiential teaching approach to develop a hands-on experience for working in the humanitarian sector. It is mandated for prospective Medair employees but open to the public. There are no restrictions to the number of participants. The main topics include an overview of Medair and understanding humanitarian aid, others and self. The cost of the course is dependent on the participant’s experience with relief work.^38^

**Training Assistance Program (TAP)** 19: This training program is similar to the CHART program. The difference is that the host organization helps with the designing of the curriculum and develops a draft agenda based on the training objectives and target audience.^32^ The main topics are similar to CHART, and the class size is not outlined.

**United Nations Humanitarian Civil-Military Coordination (UN-CMCoord) Course and Field Course** 32: The objective of these courses is to equip humanitarian and military personnel with the skills and knowledge necessary to communicate and effectively interact with each other. Participants include governmental and non-governmental organizations, aid agencies, civil protection units, military and civil defense organizations, UN agencies and other intergovernmental bodies, the Red Cross, and Red Crescent Movement. The maximum numbers of participants for the course and field course are twenty and twenty-eight, respectively. The main topics include the roles of military and humanitarian actors in emergencies and complex emergencies. Participation in the field course is by invitation only. The prerequisites are the UN-CMCoord course and a minimum of five years of experience in civil-military coordination.^24^

**Various programs by The Salvation Army**: The goal of The Salvation Army's disaster training program is for individuals to develop the skills needed to serve during times of crises particularly in the field of mental health. Participants include spiritual care officers, disaster workers trained in critical incident stress debriefing, chaplains, and mental health professionals. There are no restrictions to the number of participants per course. The main topics include foundations of emotional and spiritual care, disaster food services, disaster social services, and incident command systems.^39^

**Various programs organized by International Critical Incident Stress Foundation (ICISF):** These programs currently offer over 40 different courses selections at conferences and trainings held around the United States. The objective is to develop the skills for handling critical incident stress and mental health issues. Participants include people working in the following fields: crisis intervention, disaster response, education, emergency services, employee assistance, healthcare, homeland security, human resources, mental health, military, spiritual care, transportation, and traumatic stress.The main topics are group crisis intervention, compassion fatigue, emotional, spiritual and psychological first aid.^40^

**World of Red Cross (WORC)**: This online general orientation program introduces the main elements of the Red Cross Red Crescent Movement. The objective is to educate participants on the history and principles of the Red Cross Red Crescent Movement, in order to improve understanding and commitment to the mandate and culture. The course is open to all Red Cross Red Crescent staff, volunteers, government authorities, donors, the media, schools, and the interested public. There are no restrictions to the number of participants per course. The main topics include the origin and history of the Red Cross. This course is one of the three prerequisite for the IMPACT program.^23^

## DISCUSSION

A successful and efficient response to international disasters and complex humanitarian emergencies depends on the capacity, skills, and prior training those responders have already acquired. As the number of complex humanitarian emergencies and disasters increase, one of the challenges facing responding organizations is to maintain well-trained volunteers and employees. There is an increasing demand for skilled health providers, public health professionals, and field program administrators; this requires a cadre of trainees with both general and specific capacities.^11^

The SPHERE standards were created in 1997 to define a set of universal minimum standards in core areas of humanitarian assistance.^41^ This project was a collaborative effort by many agencies to produce standards that relate to disaster assistance in: 1) water supply and sanitation; 2) nutrition and food aid; 3) shelter and site planning; and 4) health services.^42^ Despite the definition of the SPHERE guidelines, many organizations do not train their personnel in the applications of these guidelines.^11^

Due to the various objectives of the programs along with the various expectations and interests of the trainees, there is no single training program that is universally accepted as the ideal. The main language used for these trainings is English, but the HELP courses are taught in multiple languages. Regionalized trainings conducted in other languages such as French, Arabic, or Spanish may reduce the burden to find interpreters or obtain visas. This would reduce costs and improve logistics for participants.

The list provided in this paper offers a general description of the various training programs offered worldwide. This will help potential responders –volunteers or non-volunteers- to identify training opportunities that will build their capacities in the specific areas they are responsible for. This will allow them to consider the financial, linguistic, and logistical characteristics. With evolving technology, the responders of the future may not be limited to attending in-person training programs, but have access to virtual environment experiences. However, this modality of training is still infantile for training programs geared towards international disasters and CHE and requires a large amount of financial and technical resources. For the foreseeable future, in-person training programs remain indispensable, and they continue to grow in number, scope, breadth, location and targeted audience

The vast majority of these programs target volunteers and are known worldwide for being offered by large, reputable, and established international organizations; however many more specialized trainings exist as well. NGOs typically do staff training targeted for specific roles in the field, however these training courses are seldom published in peer-reviewed journals and may not even appear on the websites of the NGOs who offer them. A centralized database of all training programs would be beneficial.

Limitations

There is a paucity of peer-reviewed literature with substantial information on structured training programs for responders to international disasters and CHE. Twelve articles on specific training programs were published in peer-reviewed journals. However, it is important to note that the literature search was not conducted in a systematic fashion due to resource constraints. There is no single web-based resource that is comprehensive, robust, and well known enough that potential responders can consider. In addition to the programs reported and analyzed in this report, there are many other smaller programs with different foci.

Additionally, there is no published literature on the recommended consensus-based recommendations for competencies or curricula. Future work could include surveying participants on their experiences in each program.

## CONCLUSIONS

Training programs available for responders to international disasters and CHE vary in their objectives, target audiences, modules, geographical locations, and financial cost. Currently, there is no resource that centralizes the descriptions of humanitarian response training programs. This paper presents an overview of available programs and serves as a resource for potential responders and organizations interested in capacity-building training prior to an international disaster response or complex humanitarian emergency.
